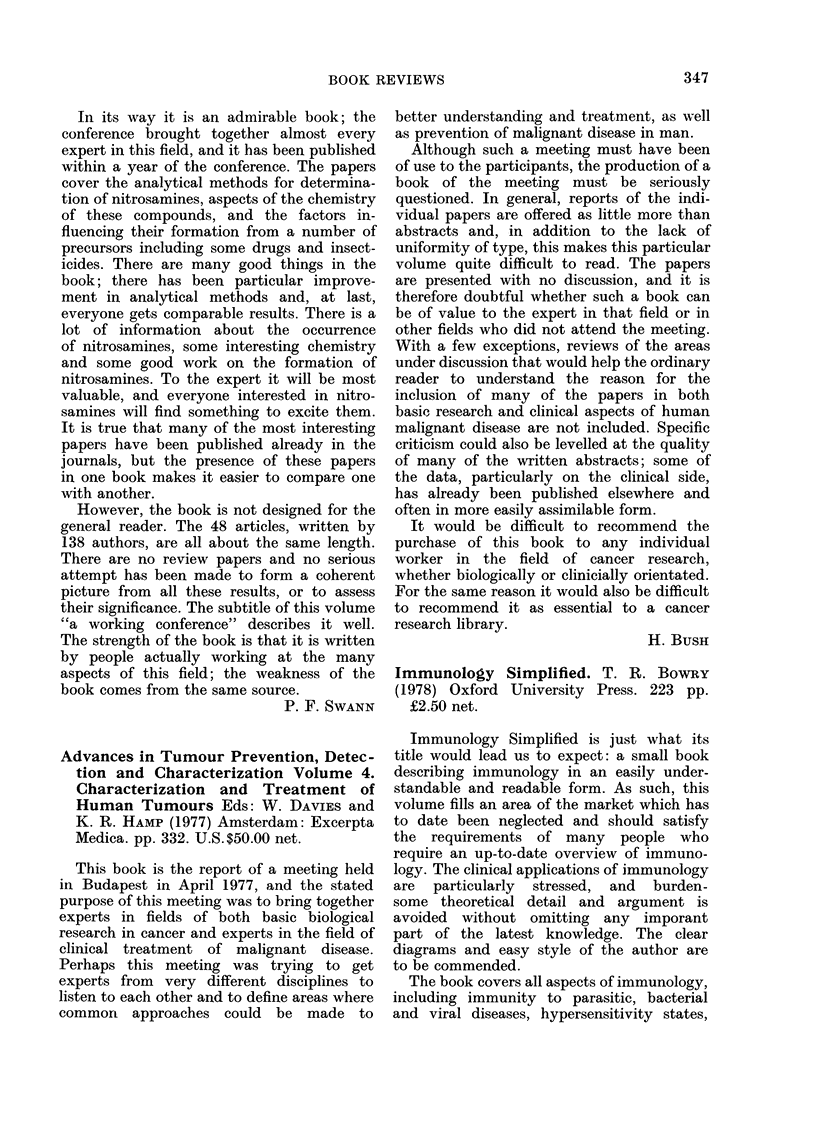# Advances in Tumour Prevention, Detection and Characterization Volume 4. Characterization and Treatment of Human Tumours

**Published:** 1979-03

**Authors:** H. Bush


**Advances in Tumour Prevention, Detection and Characterization Volume 4. Characterization and Treatment of Human Tumours** Eds: W. DAVIES and K. R. HAMP (1977) Amsterdam: Excerpta Medica. pp. 332. U.S.$50.00 net.

This book is the report of a meeting held in Budapest in April 1977, and the stated purpose of this meeting was to bring together experts in fields of both basic biological research in cancer and experts in the field of clinical treatment of malignant disease. Perhaps this meeting was trying to get experts from very different disciplines to listen to each other and to define areas where common approaches could be made to better understanding and treatment, as well as prevention of malignant disease in man.

Although such a meeting must have been of use to the participants, the production of a book of the meeting must be seriously questioned. In general, reports of the individual papers are offered as little more than abstracts and, in addition to the lack of uniformity of type, this makes this particular volume quite difficult to read. The papers are presented with no discussion, and it is therefore doubtful whether such a book can be of value to the expert in that field or in other fields who did not attend the meeting. With a few exceptions, reviews of the areas under discussion that would help the ordinary reader to understand the reason for the inclusion of many of the papers in both basic research and clinical aspects of human malignant disease are not included. Specific criticism could also be levelled at the quality of many of the written abstracts; some of the data, particularly on the clinical side, has already been published elsewhere and often in more easily assimilable form.

It would be difficult to recommend the purchase of this book to any individual worker in the field of cancer research, whether biologically or clinicially orientated. For the same reason it would also be difficult to recommend it as essential to a cancer research library.

H. BUSH